# Notice

**Published:** 1889-06

**Authors:** W. S. Kendrick

**Affiliations:** Executor Estate of A. B. Ashworth, dec’d.; Atlanta, Ga.


### NOTICE.

The death of the Proprietor and Business Manager of the JOURNAL, Dr. A. B. Ashworth, will in nowise disturb its future publication.

The JOURNAL will be issued regularly as before, and no effort will be spared, under the new management, to make its pages even more interesting and attractive with each succeeding issue in the future.

All contracts with subscribers, advertisers or others will be faithfully and fully carried out.

W. S. KENDRICK,
Executor Estate of A. B. Ashworth, dec'd.

*Atlanta, Ga., May 31, 1889.*

